# The diversity and quality of forages and their potency as herbal anthelmintic for swamp buffalo in Brebes District, Central Java

**DOI:** 10.14202/vetworld.2023.1496-1504

**Published:** 2023-07-19

**Authors:** Fadjar Satrija, Nanis Nurhidayah, Dewi Apri Astuti, Elok Budi Retnani, Sri Murtini

**Affiliations:** 1 Division of Parasitology and Medical Entomology, School of Veterinary Medicine and Biomedical Sciences, IPB University, Bogor, Indonesia; 2 Research Center for Veterinary Science, National Research and Innovation Agency, Indonesia; 3 Department of Animal Nutrition and Feed Technology, Faculty of Animal Science, IPB University, Bogor, Indonesia; 4 Division of Medical Microbiology, School of Veterinary Medicine and Biomedical Sciences, IPB University, Bogor, Indonesia

**Keywords:** forages, herbal anthelmintics, Indonesia, nutrition, pastoral, swamp buffalo

## Abstract

**Background and Aim:**

Swamp buffaloes play an important role in the rural economy of Indonesia. They consume various forages during their grazing time, including those with anti-parasitic potential. However, the information about the type and quality of forages and their potential as a natural anthelmintic for swamp buffalo is very limited. This study aimed to identify the diversity, quality, and anthelmintic potency of forages consumed by swamp buffaloes reared in Bantarkawung Subdistrict, Brebes District, Central Java Province, Indonesia.

**Materials and Methods:**

Samples of forages were obtained during three observation periods of the study, with a 12-week interval between each period. Forage diversity was evaluated by identifying its consumption by swamp buffaloes during their grazing activity in the field and feeding time in their shed. The quality of forages was analyzed using proximate analysis to measure their dry matter (DM), crude fiber (CF), crude protein (CP), crude fat (CFat), and ash contents. This is followed by the calculation of their total digestible nutrient based on the proximate analysis results. Botanical composition analysis was then conducted to measure the predominance of forages consumed by the livestock during their grazing activity. Literature reviews were carried out to explore forage’s anthelmintic activity.

**Results:**

The results showed that swamp buffaloes consume nine species of forage in the shed and 47 in the grazing area, including nine legumes, 18 grass, and 20 others. Swamp buffaloes consumed forages of lower quality, which contained high CF contents and varying levels of other nutrients below their daily nutritional needs. The grazing activity allowed swamp buffaloes to consume a higher variety of forages with better nutritional quality, thereby enabling them to meet their nutritional needs. Legumes and other forages served as the major protein sources, providing CP of 20.03% DM and 11.53% DM, and CF levels of 17.01% DM and 20.35% DM, respectively. The results also showed that the consumption of these forages increased during the rainy season. The predominant species of legumes consumed were *Leucaena leucocephala* and *Acacia* spp., while *Alternanthera sessilis* and *Merremia umbellata* were the predominant species of other forages. A total of 13 of the 47 species could potentially be used as natural anthelmintic due to their secondary metabolites, namely, tannin, flavonoid, saponin, terpenoid, diterpenoid, and mimosine. These compounds exert anthelmintic effects by inhibiting egg-hatching and larval development, as well as damaging the surface structure of both larvae and adult worms, ultimately leading to the death of the parasites.

**Conclusion:**

Overall, swamp buffaloes consumed more variety of forages during grazing compared to when they were kept in sheds. While the low-variety and low-quality forages provisioned for swamp buffaloes in their shed resulted in a low nutrient intake below their daily requirement. Furthermore, daily grazing activities allowed swamp buffaloes to fulfill and supplement their need by consuming a variety of grasses, legumes, and other forages in their respective grazing areas. Some of these forages also have the potential to become natural anthelmintic because they contain secondary metabolites, such as tannins, flavonoids, saponins, terpenoids, diterpenoids, and mimosine.

## Introduction

Swamp buffalo, also known as *Bubalus bubalis*, plays important roles in the rural economy of Indonesia as a protein source, draught animal, source of organic fertilizer, and family savings. Brebes District is one of the major swamp buffalo-raising areas in Central Java Province, Indonesia. A total of 7469 swamp buffaloes were raised in Brebes District in 2019 with the highest concentration located in Bantarkawung Subdistrict, which had a swamp buffalo population of 1419 [1].

Swamp buffalo farmers in Brebes apply a semi-intensive system combining extensive daily grazing with intensive (in-shed) feeding at night using cut-and-carry forages. This livestock-raising system has been applied for a long time by cattle and buffalo farmers on Java Island. The implementation of this particular system is influenced by the economic, social, and cultural factors within the local community, which primarily engage in rice farming [2].

Epidemiological studies on swamp buffalo parasites in Brebes District showed that a high prevalence of helminth infections with low infection intensity occurred within the population. The risk factors for parasite infection arise from the host’s internal and external aspects, such as the age and sex of swamp buffaloes, hay feeding, and the intensity of livestock health checks [3, 4].

The nutritional aspect is a well-known factor for the resistance and resilience of ruminants against helminthiasis [5]. Some important parts of the integrated helminth control program are improving livestock nutrition, proper grazing management, and genetic selection [6]. Furthermore, the provision of various Indonesian local forages for ruminants has been shown to have anthelmintic properties due to ingredients that can harm parasitic worms [7–10].

Swamp buffaloes consume various forages during their grazing time, including those with anti-parasitic potential. The information about the type and quality of forages and their potential as a natural anthelmintic for swamp buffalo in Brebes District are not yet available. Therefore, this study aimed to identify the variety of forages consumed by swamp buffaloes, analyze their quality on their nutrient content, measure the nutrient intake of swamp buffaloes in their shed, and conduct literature reviews to identify forages that have the potential as a natural anthelmintic for swamp buffalo in Bantarkawung Subdistrict, Brebes District, Central Java Province, Indonesia.

## Materials and Methods

### Ethical approval and Informed consent

This study obtained ethical approval from the Animal Ethics Committee, Institute of Research and Community Development, IPB University with registration No. 133-2018 IPB. Appropriate informed consent was obtained to collect animal stool samples, while animal sampling was performed using an approved protocol.

### Study period and location

The study was conducted from December 2018 to May 2019 in the Bantarkawung Subdistrict, Brebes District, Central Java Province. This location was selected because it had the highest swamp buffalo population in Brebes District.

### Study design

This exploratory study involved identifying forage varieties, analyzing their quality, measuring livestock consumption, and literature reviews. The implementation of the study activities was divided into three observation periods: the early, middle, and late with a 12-week interval between each period. These observation periods were determined based on the climatological data obtained from the Meteorology, Climatology, and Geophysics Agency Semarang Climatology Station and carried out in December 2018, February 2019, and May 2019, respectively.

A total of 13 swamp buffaloes that belong to the Kebandungan Livestock farmer group members, were observed in this study. The average body weight (BW) of swamp buffaloes during the early, middle, and late observation periods of the study was 280.80 kg, 299.95 kg, and 317.61 kg, respectively. These swamp buffaloes were kept in a colony shed owned by a farmer group in Kebandungan Village and released daily to graze for 8 h in the shared grazing areas. Feed consumption levels were measured during the early, middle, and late observation periods by monitoring the amount of feed consumed by swamp buffaloes during their stay in the shed from night to morning (6.00 pm–10.00 am). Feed consumption level was measured in all 13 swamp buffaloes for 7 consecutive days.

### Forages identification

Samples were collected from the provisioned forages in the shed, including all stems, roots, leaves, and flowers. Other samples were collected from grazing areas of swamp buffaloes, such as around the teak forest, natural grasslands in the forest, riverbanks, and rice fields. The grazing trail of swamp buffalo population was tracked, which started at 10.45 am and finished around 7.45 pm. On average, swamp buffaloes covered 8.71 km/day with a movement speed of 2.09 km/h. They grazed between 197 and 215 m above sea level (masl). Forage sampling was carried out in 50 spots (quadrants) measuring about 50 cm × 50 cm, separated by 200 m from one spot to another [11].

The collected forages were placed in plastic bags to be weighed and sorted based on the dominance of species. Forage samples were then turned into a herbarium for the identification process, which was conducted by the Agrostology Laboratory, Faculty of Animal Husbandry, IPB University. The identification process was carried out down to the species level and based on the guidelines [12]. Forages that could not be identified using the said guidelines were then re-identified using the identification key available on the PlantNet android application.

The identified forages from both buffalo sheds and grazing areas were grouped into three, namely, legume (Family *Fabaceae*), grass (Family *Poaceae*), and other forages [13]. Samples of the two most dominant species in each group were then cut into pieces and weighed to prepare a 150 g proximate analysis of each species. The proximate analysis, which measures crude protein (CP), fiber, and fat and the ash content of forage samples, was conducted at the Laboratory of Forest Biotechnology Research Center for Biological Resources and Biotechnology, IPB University.

### Measurement of nutrient intake

Daily consumption of fresh feed per observation period was calculated by subtracting the provisioned feed amount from the leftover amount. The provisioned feed was weighed before being given to the livestock, while the leftover was weighed the next morning at the same time. The observed variables include the daily intake of dry matter (DM), crude fiber (CF), CP, crude fat (CFat), nitrogen-free extract (NFE), and total digestible nutrients (TDN), which were calculated using the following formula [14]:

DM intake = fresh feed consumption × DM content

CP intake = DM intake × feed CP content

CFat intake = DM intake × feed CFat content

CF intake = DM intake × feed CF content

Total digestible nutrients of rice and corn straw (%) = 92.464 – 3.338 (CF) – 6.945 (CFat) – 0.762 (NFE) + 1.115 (CP) + 0.031 (CF2) – 0.133 (CFat2) + 0.036 (CF) (NFE) + 0.207 (CFat) (NFE) + 0.100 (CFat) (CP) – 0.022 (CFat2) (CP).

Total digestible nutrients of fresh grass and forages (%) = –54.572 + 6.769 (CF) – 51.083 (CFat) + 1.851 (NFE) – 0.334 (CP) – 0.049 (CF2) + 3.384 (CFat2) – 0.086 (CF) (NFE) + 0.687 (CFat) (NFE) + 0.942 (CFat) (CP)– 0.112 (CFat2) (CP).

Total digestible nutrients of legumes (%) = –133,726 – 0.254 (CF) + 19,593 (CFat) + 2,784 (NFE) + 2,315 (CP) + 0.028 (CF2) – 0.341 (CFat2) – 0.008 (CF) (NFE) – 0.215 (CFat) (NFE) – 0.193 (CFat) (CP)+ 0.004 (CFat2) (CP).

### Literature review

Literature reviews were conducted to obtain information on forages with the potential as a natural anthelmintic for livestock, especially swamp buffalo, in the study location. Reference sources were selected from scientific publications in national and international journals. The collected information comprised the genus/species, effective plant parts, secondary metabolites, and their effects on worms in both *in vitro* and *in vivo* experiments conducted on these plants.

### Statistical analysis

The data were analyzed descriptively using Microsoft Excel (Microsoft 365, Washington, USA) and presented as tables and graphs.

## Results

### Variety of forages consumed by swamp buffalo

#### Variety of forages consumed by swamp buffalo in the shed

Swamp buffaloes only consumed one type of forage during the early observation period, namely, rice straw, with the botanical name *Oryza sativa* from Family *Poaceae*, which is an agricultural byproduct. During the middle observation period, swamp buffaloes only consumed another type of agricultural byproduct in the form of corn straw (*Zea mays*) from Family *Poaceae*. During the late observation period, swamp buffaloes consumed seven species of field grass from Family *Poaceae*, namely, *Chrysopogon aciculatus*, *Cynodon dactylon*, *Eleusine indica*, *Oplismenus compositus*, *Fimbristylis alboviridis*, *Cyperus difformis*, and *Digitaria nuda*.

#### Variety of forages consumed by swamp buffalo during their grazing time

Swamp buffaloes consumed three types of legumes, nine grasses, and two other forages during the early observation period. The variety of consumed forages increased during the middle observation period to seven types, nine, and 15 types of legumes, grasses, and others, respectively. During the late observation period, swamp buffaloes consumed three types of legumes, nine grasses, and nine other forages (Table-1).

**Table-1:** The variety of forages consumed by swamp buffaloes during their grazing time in Bantarkawung Subdistrict, Brebes District, Central Java.

Groups	Family	Species name	Observation period			Local name

			Early	Middle	Late	
Legumes	*Fabaceae*	*Acacia* spp.	√	√	√	Akasia
		*Bauhinia* spp.	–	√	–	Bunga kupu-kupu
		*Centrosema molle*	–	–	√	Kacang sentro
		*Centrosema pubescens*	√	–	–	Kacang sentro
		*Desmodium triflorum*	–	√	–	Jukut Jarem
		*Gliricidia sepium*	–	√	–	Glirisidi
		*Leucaena leucocephala*	√	√	√	Lamtoro
		*Mimosa invisa*	–	√	–	Ki kerbau
		*Mimosa pudica*	–	√	–	Putri malu
Grass	*Poaceae*	*Axonopus compressus*	√	–	√	Jukut pahit
		*Bambuseae*	√	√	–	Bambu
		*Brachiaria paspaloides*	–	–	√	*
		*Chrysopogon aciculatus*	√	–	–	Rumput jarum
		*Cynodon dactylon*	√	√	√	Rumput grinting
		*Cyperus difformis*	√	√	–	Rumput teki
		*Digitaria nuda*	√	√	√	Rumput lapangan
		*Echinochloa colona*	–	√	–	Rumput bebek
		*Eleusine indica*	√	–	–	Rumput belulang
		*Eulalia trispicata*	–	√	–	*
		*Fimbristylis alboviridis*	–	–	√	Babawangan
		*Heteropogon contortus*	√	–	–	Rumput jarum
		*Imperata cylindrica*	–	√	√	Alang-alang
		*Ischaemum indicum*	–	√	–	*
		*Oplismenus compositus*	–	–	√	Jampang kriting
		*Panicum repens*	√	–	√	Jejahean
		*Pennisetum purpureum*	–	–	√	Rumput gajah
		*Zoysia matrella*	–	√	–	*
Other Forages	*Acanthaceae*	*Andrographis paniculata*	–	√	–	Sambiloto
	*Amaranthaceae*	*Alternanthera sessilis*	√	√	√	Kremah
	*Amaranthaceae*	*Alternanthera philoxeroides*	–	√	–	Bayam dempo
	*Asteraceae*	*Mikania scandens*	–	–	√	Sembung rambat
	*Asteraceae*	*Tridax procumbens*	–	√	–	Gletang
	*Convolvulaceae*	*Ipomoea carnea*	–	√	–	*
	*Convolvulaceae*	*Ipomoea sepiaria*	–	√	–	*
	*Convolvulaceae*	*Merremia umbellata*	√	√	√	Roots slemang
	*Euphorbiaceae*	*Manihot* spp.	–	√	–	Singkong
	*Euphorbiaceae*	*Euphorbia hirta*	–	–	√	Patikan kebo
	*Gentianaceae*	*Fagraea racemosa*	–	√	–	*
	*Malvaceae*	*Sida rombifolia*	–	√	√	Otok–otok
	*Malvaceae*	*Urena lobata*	–	√	√	Pulutan
	*Melastomataceae*	*Melastoma malabathricum*	–	–	√	Harendong
	*Moraceae*	*Fatoua pilosa*	–	√	–	*
	*Phyllanthaceae*	*Baccaurea polyneura*	–	–	√	Menteng
	*Rubiaceae*	*Diodia sarmentosa*	–	√	–	Rumput keriting
	*Rubiaceae*	*Oldenlandia corymbosa*	–	√	–	Rumput mutiara
	*Salicaceae*	*Populus* spp.	–	–	√	*
	*Vitaceae*	*Leea indica*	–	√	–	Girang

√=Consumed by livestock, –=Not consumed by livestock,

* =Local name unknown

The result of the botanical composition analysis of forages in the study area showed that the dominant forages species during the early observation period came from the grass group, followed by other forages, and legumes, accounting for 53.33%, 33.33%, and 13.33%, respectively. During the middle observation period, the grass group still dominated the type of forages consumed by swamp buffaloes. However, the dominance of the legume group increased to 37.50% while the other forages decreased to 6.25%.

During the late observation period, there was a major shift of dominance. In contrast, the other forages group comprised 50.00% of consumption by swamp buffaloes, followed by the grass and legume groups, accounting for 33.33% and 16.67%, respectively. There were two dominant legume species in the grazing area of swamp buffalo, namely, *Leucaena leucocephala* and *Acacia* spp. The two dominant grass species were *C. difformis* and *D. nuda*, while the other forages species were dominated by *Urena lobata* and *Merremia umbellata*.

### Forages quality

The proximate analysis results showed that swamp buffaloes consumed low-quality forages while staying in the shed (Table-2). These forages contained a high amount of CF with low CP content. The highest CF content of 26.15% was found in field grass, followed by rice and corn straw, accounting for 23.69% DM and 19.86% DM, respectively. On the other hand, the corn straw had the highest CP content of 12.43%, followed by rice straw (9.48%) and field grass (6.13% DM).

**Table-2:** Proximate analysis results of forages samples consumed by swamp buffalo in Bantarkawung Subdistrict, Brebes District, Central Java.

Sources and types of forage	DM	CFat	CP	CF	TDN

	In 100% DM (DM bases)				
Sheds (evening’s consumption)					
Rice straw	89.01	2.06	9.48	23.69	54.60
Corn straw	78.49	0.93	12.43	19.86	54.27
Mixed Grass (*Chrysopogon aciculatus, Cynodon dactylon, Eleusine indica,* *Oplismenus compositus, Fimbristylis alboviridis, Cyperus difformis,* and *Digitaria nuda*)	59.17	1.34	6.13	26.15	57.71
Grazing area					
Mix of *Leucaena leucocephala* and *Acacia* spp.	50.08	6.17	20.03	17.01	66.03
Mix of *Cyperus difformis* and *Digitaria nuda*	49.17	1.10	9.90	25.83	60.26
Mix of *Urena lobata* and *Merremia umbellata*	58.22	1.43	11.53	20.35	64.36

Grazing in the afternoon until evening allowed swamp buffaloes to consume a wider variety of forages. The proximate analysis showed that the CP, fat, and fiber content of grass from the grazing area were 9.90% DM, 1.10% DM, and 25.83% DM, respectively. Swamp buffaloes also consume legumes, which can serve as a potential source of protein. A mixture of *L. leucocephala*, and *Acacia* spp. contains 20.03% DM CP, 6.17% DM fat, and 17.01% DM fiber. The consumption of other forages, which became more dominant toward the late observation period, helped improve quality of animal feed. This was due to the more nutritious content of this forage group which consisted of 11.53% DM CP, 1.43% DM CFat, and 20.35% DM CF.

### Nutrient intake while swamp buffaloes were in their shed

The feed nutrient intake of swamp buffaloes, when they were kept in their shed, fluctuated between each observation period, as shown in Table-3. Dry matter intake steadily decreased between each observation period from 6025.29 g/head/day to 5734.94 g/head/day. Meanwhile, the intake of CFat, CF, and TDN decreased in the middle before increasing in the late observation period. The CP intake increased from 571.32 g/head/day in the early observation period to 720.74 g/head/day in the middle observation period. This figure eventually decreased to 351.83 g/head/day in the late observation period.

**Table-3:** Nutrient intake while swamp buffaloes were kept in their shed (8.00 pm–10.00 am).

Nutrient intake (% DM)	Observation period and type of feed consumed		

	Early	Middle	Late
		
	Rice straw	Corn straw	Mixed Grass (*Chrysopogon aciculatus, Cynodon dactylon, Eleusine indica, Oplismenus compositus, Fimbristylis alboviridis, Cyperus difformis,* and *Digitaria nuda*)
DM			
(g/head/day in sheds)	6025.29	5796.18	5734.94
(g/kg MBS)	52.97	52.54	40.79
CFat			
(g/head/day in sheds)	123.88	53.91	76.57
(g/kg MBS)	1.09	0.49	0.54
CP			
(g/head/day in sheds)	571.32	720.74	351.83
(g/kg MBS)	5.02	6.53	2.50
CF			
(g/head/day in sheds)	1427.63	1151.26	1499.40
(g/kg MBS)	12.55	10.44	10.66
TDN			
(g/head/day in sheds)	3290.00	3145.51	3309.58
(g/kg MBS)	28.93	28.51	23.54

*MBS=Metabolic body size (BW^0.75^), DM=Dry matter, CF=Crude fiber, CP=Crude protein, CFat=Crude fat, TDN=Total digestible nutrient

### Anthelmintic potential of the consumed forages

Swamp buffaloes consumed 47 genera/species of forages during the study period. Based on the literature study results, 13 out of 47 consumed forages are known to contain secondary metabolites, such as tannins, flavonoids, alkaloids, saponins, and other compounds. These metabolites could negatively affect the parasites, thereby turning them into potential natural anthelmintics, as shown in Table-4 [15–33]. They are found in the leaves, stems, roots, or within the whole parts of plants. Furthermore, they cause various effects, such as decreasing the parasite egg-hatching ratio, inhibiting larvae development, and damage to the cuticle of adult worms, as well as reducing the adult worm development ratio within the host body. Most studies on the benefits of forages as natural anthelmintics were conducted in developing countries. Therefore, they could potentially be developed in Indonesia as a solution to the problem of the availability of chemical anthelmintics in remote areas.

**Table-4:** Results of forages literature searches and their efficacy as anthelmintic.

Name of genus/species	Plant part	Active ingredients	Effects on parasite and targets	References
*Acacia* spp.	Pods	Flavonoids	Ovicidal and larvicidal activities against egg and larva of *Haemonchus contortus*	[15]
	Leaf	-	Adult *Haemonchus contortus*	[16]
*Bauhinia* spp.	Ethanolic extract	-	Adult *Ascaridia galli*	[17]
*Euphorbia hirta*	Whole plant	Flavonoid, Tannin, Alkaloid	*in vitro* antimicrofilarial *Onchocerca volvulus*	[18]
	Crude Methanolic ectract	-	Egg laying capacity and larvacidal of nematodes in goat	[19]
*Gliricidea sepium*	Leaf	Flavonol oxytroside	Anti-exsheatment activity of *Cooperia punctata*	[20]
*Leucaena leucocephala*	Leaf extract	Mimosine	Head thrashing, egg-laying, and mean pump amplitude of pharyngeal pumping activity of *Caenorhabditis elegans*	[21]
	Leaf extract	Tannins and flavonoids	Inhibition activity in the hatching of trichostrongylid eggs	[22]
*Mimosa pudica*	Methanolic leaf extract, *in vitro*	-	*Heligmosomoides bakeri*	[23]
	Ethanol leaf extract, *in vitro*	-	Adult *Ascaridia galli*	[24]
*Cynodon dactylon*	Whole plant	Glikosida, Tannin, Alkaloid, Resin	Gastrointestinal nematodes of goats and sheep	[25, 26]
*Alternanthera sessilis*	Whole plant	Tannin, Saponin, Terpenoid, Flavonoid	*Haemonchus contortus*	[27]
*Andrographis paniculata*	Leaf, Stems, Seed	Diterpenoid, Flavonoid	Ovicidal and Larvicidal *H. contortus* in goat and sheep	[28, 29]
*Manihot* spp.	Leaf	Tannin, Terpenoid	Ovicidal and Larvicidal *H. contortus* in goat	[30]
*Melastoma malabathricum*	Leaf	Tannin, Flavonoid	Larvicidal *H. contortus* in goat	[31]
*Populus* spp.	Stems	Flavonoid	Sheep’s Nematoda	[32]
*Urena lobata*	Powder and extract of leaf, *in vitro*	-	Adult tapeworms in chicken	[33]

## Discussion

Swamp buffalo in the study area consumed low-quality and relatively less varied forages while being kept in their shed. The main sources of their feed are agricultural byproducts, such as rice or corn straw, and a mixture of various types of grass without the addition of concentrate. The same conditions were found in most swamp buffalo farms in Southeast Asia [34, 35]. Furthermore, the feed consumed by the livestock in their shed contained high CF and low CP content. Crude fiber is a non-water-soluble plant fiber that cannot be hydrolyzed by strong acids or bases for 30 min. Its content is generally used as a reference for feed fiber, as it comprises 0.2–0.5 parts of the total fiber content of a feed ingredient. Meanwhile, high CF content marked a low feed quality [36].

The low feed quality in swamp buffaloes shed results in low nutrient intake, even below their daily requirement, as shown in Table-3. Asian swamp buffalo needs 27.00–29.78 g TDN/kg metabolic body size (MBS/BW^0.75^) per day for maintenance [37]. During the growth and lactation phases, TDN requirement of swamp buffalo increased to 27.50–52.00 g TDN/kg MBS and 35.30 g TDN/kg MBS, respectively. The daily protein requirement for Asian swamp buffalo is between 3.12 and 5.14 g CP/g weight gain for maintenance and 0.46 and 0.46–0.60 g CP/g weight gain for the growth phase [38]. On the other hand, DM intake requirements for the growth and lactation phases of swamp buffalo ranged from 2.20% to 3.15% BW and 2.50%–3.25% BW, respectively [39].

In this study, swamp buffaloes were allowed to graze in the afternoon until the evening, enabling them to consume various forages and supplement their nutritional needs. Consumption of these high-protein feeds would increase the daily nutritional intake, particularly during periods of compromised health, such as helminthiasis. These feeds can also improve digestive tract functions and effectiveness and provide essential protein and amino acids for tissue recovery and additional energy sources [5, 40, 41]. The consumption of high-protein diets also increased the value of several blood parameters related to tissue defense and immunity-building processes, such as eosinophils, hematocrits, and hemoglobin [42]. Therefore, swamp buffalo that consumes high-protein feed tends to reduce the impact of infection and show a good productivity performance as found in swamp buffaloes in the study area.

The type of feed consumed is correlated with the occurrence of helminthiasis because some forages have secondary metabolites containing anthelmintic properties, such as tannins, flavonoids, saponins, and mimosines. These substances have anthelmintic effects on animals and humans. Some legumes found in this study, such as *L. leucocephala*, *Acacia* spp., and *Gliricidia sepium* are known to be tannin-rich forages [15, 16, 20, 21]. Tannins are one of the most-studied secondary metabolites for their role as natural anthelmintics, especially in small ruminants [43]. They are the most abundant polyphenolic compounds in plants, consisting of hydrolyzed (hydrolyzable tannin) and condensed (condensed tannin [CT]) fractions. Hydrolyzable tannin fraction consists of the esters of gallic acid and ellagic sugar, which are easily soluble in acids, alkalis, hot water, and enzymes. Meanwhile, CT fraction is a polyphenol with high molecular weight consisting of oligomers and polymers of various flavan-3-ol monomer units, such as catechins, epicatechins, and others. They cause a stronger anthelmintic effect due to their ability to bind to proteins in the cuticle, oral orifice, esophagus, cloaca, and vulva of nematodes and change their physical and chemical properties. In addition, tannins can bind with feed proteins, thereby saving them from ruminal degradation [44].

Other local forages such as *Acacia* spp., *Bauhinia* spp., *L. leucocephala*, and *Manihot* spp. can reduce the hatchability of eggs *Haemonchus contortus* by 61.48%–79.15% [45]. The anthelmintic effect observed in small ruminants when fed certain plants is linked to their hydrolyzed tannin content. Studies have demonstrated that a high CT diet (30–40 g/kg DM) can also induce anthelmintic effects in these animals [46]. Condensed tannin extract negatively affected the parasites by reducing their egg-hatching ratio and damaging the cuticle structure on the surface, digestive tract, and reproductive organs of both larvae and adult worms, resulting in the death of the parasites [47]. A clinical trial has been conducted to measure the effect of CT on gastrointestinal nematodes infection, as well as the chemical and hematological parameters of infected lambs. The CT-treated group had significantly lower mean total fecal egg counts and higher blood parameters, such as hemoglobin, packed cell volume, total protein, albumin, and globulin, than the control group. Another positive impact of tannin consumption is the improvement of immune cell-mediated and humoral immune responses in lambs [48]. However, it is important to consider the effect of a tannin-rich diet on ruminants before administering them, as this will ensure that a safe and effective dosage is formulated [49].

Flavonoids and saponins are other secondary metabolites that can be found in various plants and act as anthelmintics [49]. Some flavonoids, such as anthocyanins, flavones, flavonols, flavonoids, and isoflavonoids, are the benzo-l-pyrone derivatives commonly found in fruits, vegetables, nuts, and seeds that have anti-inflammatory, antioxidant, and antimicrobial properties [50]. Anthelmintic effect of *Digitaria insularis* is determined by the content of two flavones, namely, tricin and diosmetin [51]. The previous studies by Santos *et al*. [52], Maestrini *et al*. [53] have shown that aescin and digitonin prevent egg-hatching and inhibit larval motility up to >90% and 100% egg hatch inhibition *in vitro*. The role of saponins as anthelmintic is related to their ability to form complexes with cell membrane components resulting in the formation of holes in the surface cell of worms, thereby increasing the permeability of the membranes. Several other types of saponins, such as quercetin, rutin, and epicatechin, have high efficacy against larvae *H. contortus* [54, 55].

The extent of the grazing area allows the livestock to exhibit natural behaviors, such as dung avoidance, dung heap formation, and avoidance of domination by another animal sharing the same grazing area [56–58]. Dung avoidance is a behavior in which livestock avoid foraging around the mound of feces. On the other hand, a dung heap is a specially designated area for defecation which is made by swamp buffaloes. A female swamp buffalo aged about 2 years has an average frequency of defecation of 5 times a day with 0.27 kg dry weight, covering an area of 0.14 m^2^ [59]. Furthermore, mounds of feces serve as an accumulation site, which protects the population from the pre-parasitic stage of helminths. This dung avoidance behavior will reduce the number of infective larvae or eggs ingested by livestock when consuming forages, thereby lowering the infection intensity [60].

## Conclusion

Overall, swamp buffaloes consumed nine types of grass while being kept in their sheds, and 47 types of forages when grazing, consisting of 9, 18, and 20 types of legumes, grasses, and other forages, respectively. Dry matter, CFat, CP, CF, and TDN intakes of swamp buffaloes while being kept in their sheds overnight were recorded at 6025.29, 123.88, 571.32, 1427.63, and 3290.00 g/head/day during the early observation period. Records for the middle observation period are 5796.18, 53.91, 720.74, 1151.26, and 3145.51 g/head/day while the late observation period is 5734.94, 76.57, 351.83, 1499.40, and 3309.58 g/head/day. Furthermore, the low-variety and low-quality grasses provided for swamp buffaloes in their shed caused a low nutrient intake, below their daily requirement. Fulfillment and supplementation of required nutrients were obtained from the consumption of grasses, legumes, and other forages from the grazing area in afternoon until evening. The literature review showed that 13 of the 47 types of consumed forages have the potential to become natural anthelmintics. This is because they contain secondary metabolites, such as tannins, flavonoids, saponins, terpenoids, diterpenoids, and mimosine. Conclusively, these compounds exhibit anthelmintic effects by inhibiting egg-hatching and larval development, as well as damaging the surface structure of both larvae and adult worms, ultimately resulting in the death of the parasites.

## Authors’ Contributions

FS, NN, DAA, EBR, and SM: Designed the study. FS: Performed data analysis and interpretation and drafted and revisedthe manuscript. NN and DAA: Collected and examined forage samples. EBR and SM: Co-supervised the laboratory examination. All authors have read, reviewed, and approved the final manuscript.
